# Effect of 2 sex-sorting time schedules on SIT facility management

**DOI:** 10.1093/jisesa/iead060

**Published:** 2023-09-18

**Authors:** Marco Malfacini, Arianna Puggioli, Fabrizio Balestrino, Marco Carrieri, Maria Luisa Dindo, Romeo Bellini

**Affiliations:** Department of Agricultural and Food Sciences, University of Bologna, Viale Fanin, 42, 40127 Bologna, Italy; Department of Medical and Veterinary Entomology, Centro Agricoltura Ambiente “G. Nicoli”, Via Sant’Agata 835, 40014 Crevalcore, Italy; Department of Medical and Veterinary Entomology, Centro Agricoltura Ambiente “G. Nicoli”, Via Sant’Agata 835, 40014 Crevalcore, Italy; Department of Medical and Veterinary Entomology, Centro Agricoltura Ambiente “G. Nicoli”, Via Sant’Agata 835, 40014 Crevalcore, Italy; Department of Medical and Veterinary Entomology, Centro Agricoltura Ambiente “G. Nicoli”, Via Sant’Agata 835, 40014 Crevalcore, Italy; Department of Agricultural and Food Sciences, University of Bologna, Viale Fanin, 42, 40127 Bologna, Italy; Department of Medical and Veterinary Entomology, Centro Agricoltura Ambiente “G. Nicoli”, Via Sant’Agata 835, 40014 Crevalcore, Italy

**Keywords:** SIT, *Aedes*, mosquito control, mass rearing

## Abstract

Improvements are needed in mosquito mass-rearing to effectively implement the sterile insect technique (SIT). However, managing this technique is challenging and resource intensive. SIT relies on mass rearing, sterilization, and release of adult males to reduce field populations. Maintaining an acceptable level of female presence, who can transmit viruses through biting, is crucial. Females are also essential for facility sustainability. Sex sorting plays a vital role in the production process, and our current mechanical sorting approach aims to obtain a high number of adult males with minimal female contamination within 24 h of pupation. Utilizing protandry helps control female contamination. While the 24-h sorting period achieves desired contamination levels, it may not yield enough females to sustain breeding lines, leading to increased labor costs that impact project sustainability. By delaying the sorting procedure to 48 h, we obtained sufficient females to sustain breeding lines, achieving a balance between male production and female contamination using the automatic version of the Fay–Morlan device as the sorting tool.

## Introduction

The sterile insect technique (SIT) is a genetic control strategy that involves the large-scale production, sterilization, and release of male individuals belonging to a specific target insect species(Dyck et al. 2021). It is widely recognized as a low-impact environmental technique. Integrating SIT with conventional control methods, such as insecticide application (Larramendy and Soloneski 2012), growth regulators (Achee et al. 2019, Pleydell and Bouyer 2019), or microbial larvicides (Poopathi and Abidha 2010), holds promise as an effective tool for mosquito control.

In Italy since 2000, numerous research projects have been conducted to apply and develop SIT against *Aedes albopictus* Skuse, a Diptera species belonging to the Culicidae family (Bellini et al. 2013). This species has become well established in Italy and poses significant health risk and nuisance.

The successful implementation of the SIT relies on an efficient mass production system that can generate a sufficient number of sterile males for releases and ensure the field effectiveness of the technique. Mass production should be self-sustaining providing both sterile males without residual presence of females and females to maintain breeding lines. Achieving this requires proper sexing systems and adherence to schedule protocols, as various factors such as temperature, humidity, synchronous growth, larval density, diet, strain, or adaptation level can influence the quantity and quality of produced males (Couret et al. 2014, Sasmita et al. 2019, Mamai et al. 2020, Kavran et al. 2022, Malfacini et al. 2022).

Even sterilized, females retain their feeding and vectorial capacity (Aldridge et al. 2020, Cunningham et al. 2020), and, therefore, they must be eliminated or, in countries free from endemic arbovirus transmission, significantly reduced prior to the release of adult insects into the field. In Europe, mechanical sorting systems remain more suitable for large-scale implementation of the SIT due to current opposition to genetically modified technology.

While there are potential obstacles that can affect the effectiveness of this technique, cost reduction is undoubtedly a significant factor to consider. SIT still requires substantial labor when automation is incomplete or limited to specific activities. To support mass rearing, particularly for egg production and subsequent adult generation, it is essential to have a reliable supply of biological material directly obtained from larval rearing. Ensuring self-sustainability in mass rearing is crucial, with high and consistent mosquito egg production being necessary to sustain larval colonies. Proper management of the balance between egg and sterile male production becomes essential if a mother colony is unavailable or if egg production is suboptimal (Vreysen et al. 2007).

Managing a mother colony necessitates dedicated operators, facilities, and a separate larvae production line; therefore, scaling it up is challenging and requires substantial investment. Indeed, adopting a mother colony can help mitigate the adverse effects of colonization, such as reducing colony size, shortening the developmental period, and decreasing egg productivity (Ross et al. 2019, Hendrichs and Robinson 2021, Pudar et al. 2021). In mass rearing, the focus is typically on producing males for field release while also ensuring a sufficient population of both males and females to sustain breeding lines, thereby reducing costs. During sorting operations, the presence of females affects the residual female presence in released males and the proper composition of cages. Insufficient numbers of females lead to limited egg production, negatively impacting colony sustenance. The mechanical sex sorting method, conducted 24 h after pupation, yields sufficient males for release but lacks enough females for breeding due to protandry, where males develop earlier than females, leading to an abundance of males and a lower number of females (Mamai et al. 2020).

As efforts to improve egg production and reduce the required number of females have not been successful, alternative solutions are necessary.

Delaying the sorting process to 48 h after pupation is a potential solution that could increase female production but may have implications for male productivity and female contamination.

The study investigates the effects of sex sorting at both 24 and 48 h from the onset of pupation to assess the feasibility of maintaining male productivity, residual female presence, and female production for breeding in the contest of SIT application. The purpose was to understand whether male productivity and residual female presence, in combination with female production for breeding purposes, could be effective for SIT application and sustainment. The goal is to identify cost-effective strategies that can improve the quantity and quality of mass production.

## Materials and Methods

### Insects

In 2020, *Aedes albopictus* strain colonies were established by collecting field eggs in the Emilia-Romagna region (RER). The RER strain was reared in 80 × 80 × 20 cm mass production cages (Balestrino, 2018, IT Patent No. 102018000002696; produced by A Zeta Model Sas) under standard conditions of 28 ± 1 °C, 85% RH, and a 14:10 (L:D) photoperiod at our Experimental Module—Biological Control of the Tiger Mosquito. Each cage housed 10,000 females and 3,000 males, maintaining a sex ratio of approximately 3:1. The adults were provided with a constant supply of a 10% sucrose solution, while the females were also fed defibrinated swine blood. Eggs laid on wet wrinkled paper were collected, dried, and stored in sealed containers (Bellini et al. 2013). Larval rearing was conducted using mass-rearing rack units with FAO/IAEA mosquito mass-rearing trays (Balestrino et al. 2014), and the larvae were fed a diet consisting of 50% black soldier fly powder (Innovafeed SAS, France) and 50% fish food (KOI-Franciacorta, Italy) (Mamai et al. 2019).

### Mechanical Sex-Sorting Tools and Procedures

After 24 or 48 h from the onset of pupation, an entire unit rack, composed of 49 trays containing 13,000 larvae each, was drained, and the larvae and pupae contained were sexed using an automatic version of the Fay–Morlan separator (Guangzhou Wolbaki Biotech Co. Ltd) (Fay and Morlan 1959, Focks 1980). Sixty-one and fifty-three rearing racks were respectively sex sorted at 24 and 48 h, from the onset of pupation, thus representing the replicates (one rearing rack = one replicate).

The automatic glass plate separator is a machine composed of 2 glass sheets that are moved by a motor, allowing for the adjustment of distances between them. It features an electric power supply and a graphic interface for setting sorting parameters. The device includes a larvae and pupae blender that expels material onto the glass sheets through a supply valve. Water jets clean the glass and remove larvae, which are collected in a tank. The distances between the glass sheets are automatically adjusted for male pupae collection, with a separate tray for collection. After a washing cycle, the males are conveyed to their designated tray. The glass distances increase, causing the remaining material to flow into the collection tank, which directs the females to their dedicated tray. All sorted material is collected in trays with a fine net, while the filtered water is recycled for washing.

### Sorting Time Influence Using Automatic Fay–Morlan Separator

To achieve a larval density of approximately 2.2 larvae/ml, the eggs were directly incorporated into each rearing tray along with the larval diet, following the methodology described by Zhang et al. (2015). The eggs were placed in the rearing trays to induce hatching, and data on hatching time were recorded. Since hatching, a progressively increasing quantity of larval diet was provided on a daily basis until pupation, as outlined in the study conducted by Balestrino et al. (2014).

The sex sorting procedure, employing an automated Fay–Morlan separator, as previously described, was performed either 24 or 48 h after the initiation of pupation. This sorting process was aimed at assessing the potential impact of different sorting times on male productivity and residual female presence within the population.

Specifically, our investigation focused on evaluating the influence of delaying the sorting time to 48 h after the onset of pupation on male productivity and the occurrence of female contamination among the released males. This experimental design enabled us to determine whether the timing of sex sorting had a significant effect on these particular parameters.

### Evaluated Parameters and Statistical Analysis

In this study, each replica was represented by a single unit rack, whit every tray having consistent conditions of larval density, diet, and environmental parameters. After each mechanical sorting session, conducted using an entire rearing rack, males were counted and samples were randomly collected to asses productivity and female contamination.

The productivity yield and the female contamination at the 2 scheduled times were respectively calculated as the percentage of male pupae collected on the original number of first-instar larvae present in the rearing rack, and as the percentage of females on the total sorted pupae: $\begin{array}{lll} & Residual\;female\;presence\;(\%)\;=\; & \left(number\;of\;females/number\;of\;pupae\right)\times 100\; \end{array}$. The productivity yield, being related to the total number of larvae, was multiplied by 2, assuming an equal proportion of males and females, to obtain the male productivity yield on the total number of reared males: $\begin{array}{lll} & Male\;productivity\;yield\;(\%)\;=\;\; & (number\;of\;males/number\;of\;larvae)\times 100\times 2\; \end{array}$.

Male productivity was corrected based on the residual female presence to offer more truthful data on productivity: $\begin{array}{lll} & Corrected\;productivity\;(\%)\;=\;[(1\;-\;Residual\;female\;presence/100)\; & \times male\;productivity\;yield]\; \end{array}$.

The data were analyzed by 1-way ANOVA, with the male productivity yield and the residual female presence adopted separately as dependent variables, while sorting time was adopted as the grouping variable.

## Results

The impact of sorting time on male productivity and residual female presence showed significant results (*F*_1,112_ = 5.90, *P* < 0.05 and *F*_1,112_ = 22.44, *P* < 0.01).

When sorting was performed at 48 h from the onset of pupation, the average male productivity was 31.1 ± 1.06%. This value was significantly higher (*t*_112_ = 2.43, *P* < 0.017) compared with sorting at 24 h, where the male productive yield averaged 27.8 ± 0.88% (Table 1). Additionally, the number of females sorted using the mechanical sorter within each unit rack was consistently below 10,000 at the 24-h sorting interval.

**Table 1.** Male productivity yields and residual female percentages for the 2 sorting times

Sorting time (h)	*N*	MPY (mean ± SE)	RFP (mean ± SE)
24	61	27.8 ± 0.88a	0.59 ± 0.08a
48	53	31.1 ± 1.06b	1.08 ± 0.09b

“Sorting time” indicates the number of hours from the onset of pupation in which the sieving was carried out. “*N*” represents the replicates number representative of the number of rearing rack units tested. “MPY” is the male productivity yield percentage. “RFP” is the residual female presence percentage. “Mean ± SE” is the marginal mean ± standard error of the values obtained with the 2 sorting times. Different superscript letters within a column indicate statistical differences *P* ≤ 0.05, 1-way ANOVA.

Conversely, the sorting procedure at 48 h exhibited the highest contamination of females, with a mean residual presence of 1.08 ± 0.09%. This represented a significant increase (*t*_112_ = 4.74, *P* < 0.001) compared with the 24-h sorting, which had a female contamination rate of 0.59 ± 0.09% (Table 1). Notably, each sorted unit rack during the 48-h sorting provided a minimum of 30,000 females for breeding purposes.

## Discussion

The productivity of males was slightly higher when sex sorting was carried out at 48 h from the onset of pupation compared with sorting at 24 h. However, at the 48-h sorting time, a significant but acceptable increase in residual female presence in the released males was observed. This could be attributed to the overlapping growth curves of males and females at this stage (Carvalho et al. 2014, Puggioli et al. 2017, Mamai et al. 2020). The sorting tool retainment capacity may have been affected by the hardness of the cuticle, resulting in the incorrect retention of soft females. Additionally, during the washing cycles, the water jets may have pushed softer females, causing them to compress slightly between males (Fay and Morlan 1959, Focks 1980).

Nevertheless, sorting at 48 h proved to be productive with an acceptable sex ratio, reducing labor costs and eliminating the need to reintroduce larvae into the rearing process. This allowed for male production for field application and female production for breeding purposes. On the other hand, sorting at 24 h did not yield enough females to sustain the breeding lines, requiring the reintroduction of larvae into the unit racks. This process involved additional labor and costs, including filling the racks with water, counting and aliquoting the larvae, and adding larval diet. Another sorting had to be performed after 24 h to collect females for cage preparation, adding further labor and costs.

Sorting at 48 h increased the number of females, enabling the production of 3 cages after each sorting to maintain a constant weekly production (Mamai et al. 2020). However, this scheme presented some obstacles. Some adults had already reached the adult stage at 48 h, leading to the presence of biting females in the facility. This posed a nuisance to operators and required the use of individual protection systems to ensure their well-being. Operational and technological improvements were necessary to prevent adult emergence and protect the operators’ health.

Sorting at 24 h allowed for a 24-h delay before irradiation, ensuring pupal maturation and a residual fertility lower than 1%. However, at 48 h, some pupae had already reached the critical age for emergence, while others may have been too young for proper sterilization. Delaying irradiation by waiting 24 h would result in the emergence of many adults, since *Ae. albopictus* pupae emerge around 48 h from pupation onset (Zhang et al. 2015), leading to a loss of males and affecting SIT application thus forcing an early irradiation after the sorting. Early irradiation of some male pupae could also result in increased residual fertility, since the youngest pupae are the most sensitive to irradiation and resistance increasing as the pupal age increases. Such a phenomenon could significantly impact the feasibility of the SIT when implemented in the field (Yamada et al. 2019).

To overcome these issues, new solutions for irradiating male mosquitoes at the adult stage are being adopted and developed for *Aedes* mosquitoes (Bimbilé Somda et al. 2022, Maïga et al. 2022). The use of proper tools or dedicated containment cages would allow for the isolation of adults from pupae, enabling direct irradiation of adults.

Refrigerated conditions would be necessary during adult irradiation to prevent movement, and a high adult density would limit the number of irradiations. Adult sterilization has a lower impact on induced sterility, longevity, and male mating competitiveness in *Ae. albopictus* compared with pupal sterilization. Studies on *Anopheles arabiensis* Patton have shown that adult sterilization achieves higher levels of sterility with lower doses than pupal irradiation, suggesting that adult irradiation may maintain the better overall quality of the adults (Ndo et al. 2014). Unfortunately, existing rearing systems and irradiators do not currently meet the necessary requirements. None of the known rearing units is capable of directly recovering adult mosquitoes due to the rack-like structure of the units, which does not allow for the conveyance of adult mosquitoes.

With the current method of adult sterilization, it would be necessary to enable mosquito emergence inside devices already used for field application (Tur et al. 2022). These tools could be utilized to facilitate adult emergence prior to pupal irradiation. Once the adults have emerged, they can be chilled to immobilize them before irradiation, still maintaining refrigerated conditions. However, the capacity of the adult emergence tubes, designed to meet specific requirements, may only accommodate a few thousand mosquitoes. Consequently, numerous tubes would need to be emptied to fill the dedicated sterilization container. The manipulation of samples during this process can adversely affect adult survival and competitiveness, thereby reducing field efficacy.

Moreover, a large number of mosquitoes, once irradiated and chilled, would need to be divided into several batches to ensure the proper distribution of adults in the field. Unless specifically developed releasing systems that can operate under refrigerated conditions are created, this process poses challenges.

In summary, the current rearing systems and irradiators do not allow for direct recovery of adult mosquitoes. Alternative methods involving the use of emergence devices, chilling, and subsequent irradiation under refrigerated conditions are necessary and can help to recover a higher quatity of adult males. However, these methods require further development to address challenges such as sample manipulation, batch distribution, and the need for specialized releasing systems for refrigerated conditions.

Due to these challenges, adult irradiation remains a demanding process. As a result, pupal sterilization is often preferred for field applications where an automatic mass-rearing facility is not available. In such cases, utilizing a sex-sorting method carried out at 48 h can improve the overall feasibility of the SIT approach. This approach not only improves the production and sorting of males but also ensures the availability of females for facility sustainability, thus contributing significantly to the successful implementation of the SIT.
